# Placental mosaicism in prenatal diagnosis

**DOI:** 10.1515/medgen-2026-2007

**Published:** 2026-04-16

**Authors:** Patricia Döttelmayer, Christine Fauth

**Affiliations:** Medical University of Innsbruck Institute of Human Genetics Peter-Mayr-Str. 1 6020 Innsbruck Austria; Medical University of Innsbruck Institute of Human Genetics Peter-Mayr-Str. 1 6020 Innsbruck Austria

**Keywords:** confined placental mosaicism (CPM), true fetal mosaicism (TFM), chorionic villus sampling (CVS), placental dysfunction, non-invasive prenatal testing (NIPT)

## Abstract

Chromosomal mosaicism is a well-known phenomenon in prenatal cytogenetics and affects approximately 2 % of chorionic villus samples (CVS). The interpretation of mosaicism is challenging, and the major question is whether the abnormal cell line also affects the fetus (true fetal mosaicism, TFM). While mosaicism detected at CVS turns out to be confined to the placenta in the majority of cases, the individual risk of TFM widely varies and needs to be assessed on a case-by-case basis.

This article aims to provide an overview on the different types of mosaicism in CVS, the probability of fetal involvement, the laboratory work-up, implications for genetic counselling, and potential effects upon placental function.

It is emphasized that understanding placental mosaicism is crucial for the interpretation of results from non-invasive prenatal screening technologies, like NIPT for common aneuploidies, which are based on cell free placental DNA.

Finally, we will discuss recent findings of genomic studies which indicate that placental mosaicism extends far beyond classic chromosome aberrations.

## Introduction

Chorionic villus sampling (CVS) was introduced in the 1980s as a procedure for rapid prenatal diagnosis of chromosomal and other genetic disorders in early pregnancy [1, 2, 3]. Early experiences showed that the karyotype in chorionic villi does not always reflect the fetal karyotype, especially in cases where only direct preparation / short-term culture was analysed [3, 4]. It turned out that the major reason for these discrepancies was chromosomal mosaicism in CVS [5, 6]. Chromosomal mosaicism is defined as the presence of two or more cell lines with a different chromosomal constitution derived from a single zygote and affects approximately 2 % of chorionic villus samples [7, 8].

Over the years, a vast amount of data on mosaic findings at CVS has been studied [1, 6, 8, 9]. While the overall probability of fetal involvement is estimated to be approximately 13 % [1, 10], the individual risk of true fetal mosaicism widely varies and depends on various factors, including the type of the chromosome aberration, the specific chromosome involved and the distribution pattern of aberrant cells in different placental tissues [8, 10–12].

Knowledge about placental mosaicism is essential. In many countries, chromosome analysis on chorionic villi is still the first-line diagnostic test in pregnancies with structural abnormalities on first trimester ultrasound or an increased risk for aneuploidy after standard first trimester screening. Depending on the cohort, CVS may provide a rapid and early diagnosis of chromosome aberrations in 20–40 % of cases [13–16].

In recent years, the interest in placental mosaicism has been renewed by the widespread use of non-invasive prenatal testing for common aneuploidies (NIPT) which is based on cell-free placental DNA and hence may give false positive or false negative results related to placental mosaicism [17, 18].

## Histomorphology of chorionic villi and laboratory work-up of CVS

**Figure 1:**
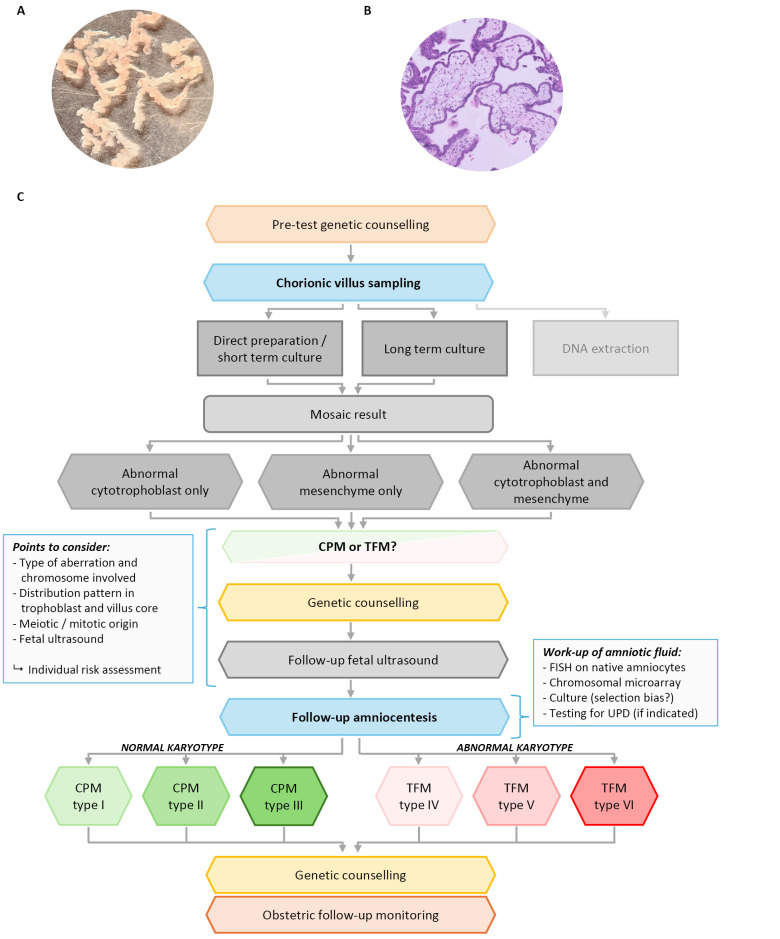
Mature first trimester chorionic villi (A) under the inverted microscope (B) histological section (HE staining). Chorionic villi are covered by trophoblast (syncytiotrophoblast and cytotrophoblast, stained in blue). The villus core contains mesenchyme with fetal blood vessels.(C) Flow diagram showing the work-up of mosaicism detected at CVS

Chorionic villi are tiny finger-like projections from the chorionic membrane that develop rapidly during early pregnancy and form the fetal part of the placenta. Mature chorionic villi (figure 1-A and 1-B) have two major layers of cells with different origin: the outer syncytiotrophoblast / cytotrophoblast (chorionic ectoderm) and the inner mesenchymal core with fetal blood vessels (chorionic mesoderm). During embryonic development, trophoblast cells are the first to differentiate from totipotent cells, while cells from the villus core differentiate later and hence are more closely related to the cells that form the embryo [19].

Full work-up of chorionic villus samples classically includes direct preparation / short-term culture and long-term culture. In direct preparation / short-term culture, spontaneously dividing cytotrophoblast cells are analysed, while cells from the mesenchymal core are analysed in long-term culture [20]. When short- and long-term culture show the same karyotype and no mosaic is found, the result is considered to reflect the fetal karyotype. 

## Origin of mosaicism and uniparental disomy

Chromosomal mosaicism arises postzygotically in mitosis. There are two principal mechanisms [21]:

(1) Mitotic nondisjunction in an initially normal zygote/embryo leads to two daughter cells with an abnormal genetic constitution – trisomy in one cell and monosomy in the other. If an autosome is affected, the monosomic cell usually is not viable and will be lost, while the trisomic cell may survive. This results in mosaicism with a normal and a trisomic cell line. If nondisjunction affects a sex chromosome, all three cell lines may be viable (e. g. 47,XXX/46,XX/45,X).

(2) Mosaicism can also arise, if an initially trisomic zygote / embryo postzygotically loses one of the supernumerary chromosomes by a mitotic error (“trisomy rescue”). This type of mosaicism involves two events: a meiotic error which leads to trisomy, followed by a mitotic error leading to the rescue of trisomy in a subset of cells. Depending on which of the three chromosomes is lost, uniparental disomy (UPD) may result. UPD is defined as the inheritance of two homologues of a chromosome from the same parent [22]. UPD which results from trisomy rescue often shows heterodisomic and isodisomic segments due to meiotic recombination [23, 24]. While the majority of UPD events probably do not lead to a clinical phenotype [23], UPD may become clinically relevant in two main ways: (1) When UPD affects a chromosome with clinically relevant imprinted genes (chromosomes 6, 7, 11, 14, 15, and 20) it may cause an imprinting disorder. (2) Recessive mutations can be unmasked by isodisomy and lead to an autosomal-recessive disease [24].

## Major categories and types of mosaicism

In prenatal diagnosis, there are two major *categories* of mosaicism: CPM – confined placental mosaicism and TFM – true fetal mosaicism. A special case is pseudo-mosaicism, which is no true mosaicism but an artifact arising in cell culture. A thorough distinction between CPM, TFM, and pseudo-mosaicism is essential.

When a mosaic chromosomal abnormality identified in CVS is confirmed at amniocentesis, it is considered TFM. CPM and TFM can be further subdivided depending on whether the chromosomal abnormality is detected in trophoblast and / or mesenchyme. Grati et al. distinguish six *types* of mosaicism in chorionic villi – three types of CPM and three types of TFM, respectively (table 1) [8].

The most frequent types are CPM I and CPM II, where the abnormal cell line is either confined to the trophoblast or to the villus core, respectively, and which together account for approximately 75 % of all cases of mosaic findings in chorionic villi.

TFM is much rarer than CPM and all types of TFM together (TFM IV-VI) account for less than 15 % of mosaic cases in chorionic villi. TFM IV and V are characterized by an abnormal cell line in trophoblast or mesenchyme, respectively, and confirmation of the same abnormality in amniocytes. In TFM VI, the chromosomal abnormality is present in trophoblast, mesenchyme, and amniocytes. 

**Table 1:** Different types of mosaicism and relative frequency (adapted from Grati et al. [8])

Mosaic type	Trophoblast	Mesenchyme	Amniocytes	Relative Frequency
**CPM I**	abnormal	normal	normal	35.73% 40.45% 10.36% 1.55% 5.36% 6.55%
**CPM II**	normal	abnormal	normal	
**CPM III**	abnormal	abnormal	normal	
**TFM IV**	abnormal	normal	abnormal	
**TFM V**	normal	abnormal	abnormal	
**TFM VI**	abnormal	abnormal	abnormal	

## Risk of fetal involvement

A huge amount of data on chromosomal mosaicism in chorionic villus samples has been collected in order to delineate the risk of fetal involvement and provide a basis for genetic counselling [1, 6, 8, 10, 11, 25].

Table 2 shows the likelihood of confirmation of mosaic findings in CVS at amniocentesis for different chromosome aberrations. The combined likelihood is based on data from four different studies [1, 6, 8, 25].

The main findings are:

Mosaicism detected at CVS is in the majority of cases restricted to the placenta (87 % CPM). The overall probability of fetal involvement (TFM) is approximately 13 % [1, 8, 10].
Autosomal trisomies are the most common type of mosaic abnormality detected in chorionic villi and account for more than half of all chromosome aberrations [8]. The overall probability of TFM for autosomal trisomies is 9.6 % (range 7.5 %–13.1 %; [1, 8]). However, the risk widely varies and critically depends on the chromosome involved. Trisomy 21 mosaicism has the highest risk of fetal involvement (40.7 %), followed by trisomies 18, 16, and 13 with a risk of 15.3 %, 10.9 %, and 7.1 %, respectively. Other autosomal trisomies with an increased risk of TFM involve chromosomes 8, 9, 20, and 22, while trisomies of chromosomes 2, 3, and 7 are mainly confined to the placenta [1, 6, 8].
Sex chromosome aneuploidies (mainly monosomy X mosaicism) and mosaic supernumerary marker chromosomes detected at CVS have the highest risk of fetal confirmation with 26.5 % and 29.7 %, respectively [8].
The likelihood of fetal confirmation in cases with mosaic structural chromosome rearrangements (unbalanced and balanced) is approximately 11 %.
The studies in table 2 mention only two cases with mosaic triploidy, both of which were confirmed at amniocentesis. In the literature, there are several case reports on mosaic triploidy confined to the placenta [26–31]. Hence, the likelihood of fetal involvement in diploid-triploid mosaicism is high, but probably less than 100 %. In contrast to triploidy, tetraploidy is a cultural artefact in the vast majority of cases [8, 32].

**Table 2:** Mosaic findings at CVS and likelihood of confirmation at amniocentesis for different chromosome aberrations

Aberration	Phillips et al., 1996 [25]	Hahnemann and Vejerslev, 1997 [6]	Grati et al., 2017 [8]	Thomsen et al., 2024 [1]	Combined likelihood of confirmation at amniocentesis
**Autosomal trisomies**	21/267 7.9 %	18/192 9.4 %	45/600 7.5 %	69/528 13.1 %	153/1587 **9.6 %**
Chromosome 13		2/15 3.3 %	1/46 2.2 %	3/23 13.0 %	6/84 7.1 %
Chromosome 16		0/11 0 %	2/22 9.1 %	8/59 13.6 %	10/92 10.9 %
Chromosome 18		4/29 13.8 %	10/57 17.5 %	8/58 13.8 %	22/144 15.3 %
Chromosome 21		9/22 40.9 %	22/60 36.7 %	19/41 46.3 %	50/123 40.7 %
**Sex chromosome aneuploidies**	17/109 15.6 %		57/170 33.5 %		74/279 **26.5 %**
45,X			34/127 26.8 %		34/127 26.8 %
47,XXX			7/13 53.8 %		7/13 53.8 %
47,XXY			7/18 38.9 %		7/18 38.9 %
47,XYY			1/3 33.3 %		1/3 33.3 %
45,X/46,XX/47,XXX			7/7 100 %		7/7 100 %
45,X/46,XY/47,XYY			1/2 50 %		1/2 50 %
**Supernumerary marker chromosomes** (47,+mar)	8/30 26.7 %		22/71 31.0 %		30/101 **29.7 %**
**Triploidy**			2/2 100 %		2/2 **100 %**
**Tetraploidy**			0/65 0 %		0/65 **0.0 %**
**Structural rearrangements** (balanced and unbalanced)	3/35 8.6 %		22/192 11.5 %		25/227 **11.0 %**
*Legend: n/N = TFM/(CPM+TFM), total numbers are given above, percentages are given below*					

The likelihood of TFM also depends on the distribution of aberrant cells in trophoblast and villus core [8, 10, 12]. When both placental layers are affected, the probability of TFM is higher than in cases where the aberrant cell line is detected in trophoblast or villus core only (36.6 % versus 3.9 % and 12.1 %, respectively) [12]. Moreover, the confirmation rate at amniocentesis significantly increases to more than 80 % when the trophoblast shows a mosaic abnormality and the villus core is homogeneously affected (non-mosaic abnormality) [8].

Furthermore, the risk of TFM is influenced by various other factors including the mechanism of formation of the mosaic aneuploidy (meiotic error with trisomy rescue versus mitotic error), the timing of the initial event leading to mosaicism during embryogenesis, and the selection for or against abnormal cells in different tissues [12, 33]. The type of origin is often chromosome-specific. While the common trisomies 21, 18, and 13 and some rarer trisomies (chromosomes 9, 15, 16, and 22) have an increased rate of meiotic origin and a higher likelihood of TFM, mosaic trisomies for chromosomes 2 and 7 mainly originate postzygotically by a mitotic error and are rarely confirmed at amniocentesis [6, 10, 12, 34].

## Genetic counselling, follow-up amniocentesis and prenatal ultrasound

A mosaic karyotype in chorionic villi requires careful genetic counselling of the parents, a detailed prenatal ultrasound and (in the vast majority of cases) amniocentesis (figure 1-C). The parents should be informed that mosaic chromosome abnormalities in placental tissue do not necessarily reflect the karyotype of the fetus and mostly turn out to be restricted to the placenta. The uncertainty inherent to chromosomal mosaicism and possible outcomes in case of TFM should be addressed. The risk of TFM needs to be assessed individually [12]. Based on published risk figures and the specific laboratory and ultrasound findings an approximate risk estimate may be offered. While the presence of ultrasound abnormalities increases the likelihood of TFM, their absence may be reassuring but does not exclude TFM [12].

A mosaic karyotype in chorionic villi usually prompts a follow-up amniocentesis. Amniotic fluid contains a heterogeneous cell population derived from the amnion and the fetus (skin, gastrointestinal, respiratory, and urogenital tract) and hence more closely reflects the karyotype in fetal tissues [20]. For work-up, a combination of different laboratory techniques is advisable: Interphase fluorescence in situ-hybridisation (I-FISH) with an appropriate probe allows for rapid screening of a large number of uncultured amniotic fluid cells for the chromosome aberration in question. Chromosomal microarray analysis on amniotic fluid can detect mosaicism at a limit of about 10–20 % [35, 36]. The sensitivity of standard karyotyping after cell culture varies and depends on in vitro selection for or against abnormal cells and the total number of cells analysed.

If the chromosome aberration is not detected in amniotic fluid, the chromosome aberration is considered to be confined to the placenta [11]. However, fetal involvement can never completely be excluded for two reasons: (1) low-level mosaicism may escape detection at amniocentesis, and (2) mosaicism may be restricted to specific fetal tissues that have no access to amniotic fluid (e. g. brain). In the literature, there are a few rare examples of fetal involvement in presumed CPM after normal amniocentesis [37, 38].

Additional UPD testing on amniotic fluid DNA should be offered when trisomy mosaicism for a chromosome with clinically relevant imprinted genes (chromosomes 6, 7, 11, 14, 15, and 20) or a small supernumerary marker chromosome (sSMC) has been detected at CVS [23, 24].

If the abnormal cell line is also found in amniotic fluid, it is considered to reflect TFM. This does not necessarily imply an adverse outcome in the fetus, as the proportion and distribution of abnormal cells in different fetal organs cannot be predicted. In the presence of specific ultrasound abnormalities, TFM poses a high risk for physical and developmental abnormalities. However, if no abnormalities are evident on ultrasound, the prognosis is unclear and genetic counselling is particularly challenging.

## False-negative results in chorionic villus sampling

False negative results in chorionic villus sampling are extremely rare [39]. In these cases, the karyotype in chorionic villi is normal, while the fetus has a chromosomal aberration (“complete feto-placental discordance”). The majority of false negative cases reported in the literature are due to incomplete work-up of chorionic villus samples, when karyotyping is based on direct preparation / short-term culture alone [6].

A rare example of complete feto-placental discordance is shown in figure 2. CVS was performed in a 32-year-old primigravida at 14+4^WG^ due to increased nuchal translucency (7.6 mm). Short- and long-term culture showed a 47,XXY karyotype in all metaphases, which could not explain the abnormal ultrasound. No additional copy number aberrations were detected at molecular karyotyping (SNP-array) on DNA extracted from cultured chorionic villi. In view of the abnormal ultrasound, the parents opted for termination of pregnancy. At autopsy, a fetal skin biopsy was taken and molecular karyotyping was repeated. Remarkably, in addition to the supernumerary X-chromosome, trisomy 21 mosaicism was found in fetal skin which provides a good explanation for the increased nuchal translucency. Fetoplacental discordance in this case is probably due to a postzygotic mitotic error affecting embryonic cells (figure 2-B).

**Figure:**
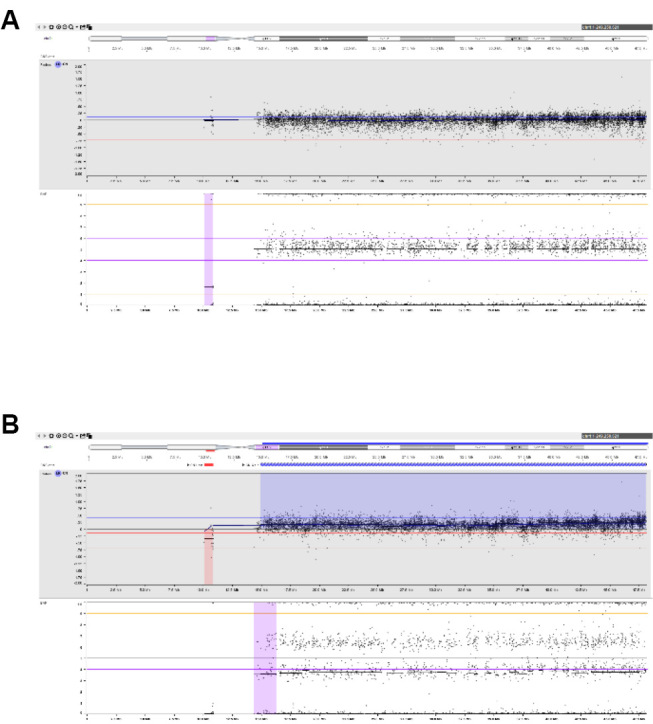
**Figure 2:** Fetoplacental discordance with a false-negative result in chorionic villi. SNP-array results for chromosome 21 on DNA extracted from (A) cultured chorionic villi and (B) a fetal skin biopsy. Log R-ratio (top) and B-allele frequency (bottom) are shown. (A) Normal result in chorionic villi. (B) Trisomy 21 mosaicism in fetal skin (~60 % trisomic cells). The pattern of B-allele frequency is consistent with a postzygotic mitotic origin of trisomy 21.

## Chromosomal mosaicism and placental function

Even when mosaicism is confined to the placenta, there may be an increased risk for adverse pregnancy outcomes. Several studies have shown that CPM for autosomal trisomies may lead to placental insufficiency with an increased risk for preeclampsia, fetal growth restriction, and preterm birth [1, 34, 40–44]. However, risk scores widely vary and may depend on the chromosome involved, the level of mosaicism, the type of CPM (increased risk particularly in CPM type III), and the presence of UPD [42, 45, 46]. Trisomy 16 mosaicism (CPM and TFM) is associated with the highest risk for pregnancy complications [41], but an increased risk has also been reported for other trisomies [1, 47, 48].

Non-mosaic trisomy 16 is the most common trisomy in spontaneous first trimester pregnancy losses and considered incompatible with life [33, 49, 50]. It typically arises from nondisjunction in maternal meiosis I. In up to 10 % of cases trisomy rescue may occur leading to mosaic trisomy 16 [33, 50]. The distribution and proportion of trisomic cells in extraembryonic and embryonic tissues influences the clinical outcome which covers a broad spectrum ranging from a normal pregnancy to fetal growth restriction, preeclampsia and preterm delivery, various fetal malformations, or even pregnancy loss [50, 52–54]. Low maternal serum levels of pregnancy-associated plasma protein A (PAPP-A) have been reported to be associated with an increased risk of adverse outcomes in pregnancies with trisomy 16 CPM [53, 55]. Hence, thorough obstetric monitoring for preeclampsia and fetal growth restriction linked to placental dysfunction is indicated [56].

Remarkably, in a study on obstetric and long-term outcomes in a cohort of children with prenatally detected CPM or TFM for trisomy 16, more than 80 % of school-aged children had a favourable long-term neurodevelopmental outcome and attended mainstream classes [50].

An example of fetoplacental trisomy 16 mosaicism is given in figure 3. A 34-year-old woman underwent chorionic villus sampling at 13+4^WG^ because of fetal tachycardia, ventricular septal defect, crown-rump length below the 5^th^ centile, and abnormal serum biochemistry (low PAPP-A: 0.097 MoM). Conventional chromosome analysis failed due to low mitotic index in direct preparation / short-term culture and poor cell growth in long-term culture. SNP array analysis on DNA extracted from native chorionic villi and long-term culture showed an apparently non-mosaic trisomy 16 in native villi and trisomy 16 mosaicism (~20 % trisomic cells) in long-term culture, respectively (figure 3-A). It should be noted that the percentage of trisomic cells in DNA extracted from long-term culture may have been distorted by selection in cell culture and does not necessarily reflect the percentage of trisomy 16 in native cells from villus core.

The couple opted for termination of pregnancy without prior amniocentesis. At autopsy, tissue biopsies for molecular karyotyping were taken from fetal skin, liver, brain, and heart. Trisomy 16 mosaicism was confirmed in fetal heart but absent in other organs (figure 3-B). Genotype analysis (based on B-allele frequency in placental / fetal tissues and comparison to parental genotypes) pointed to an origin of trisomy 16 in maternal meiosis I. Surprisingly, B-allele frequency indicated normal biparental inheritance of chromosome 16 in disomic cells in long term culture (villus core), but maternal UPD 16 in fetal tissues. This suggests that trisomy rescue occurred twice by two independent events (figure 3-C).

**Table:** 

**A **		**C **	
native villi 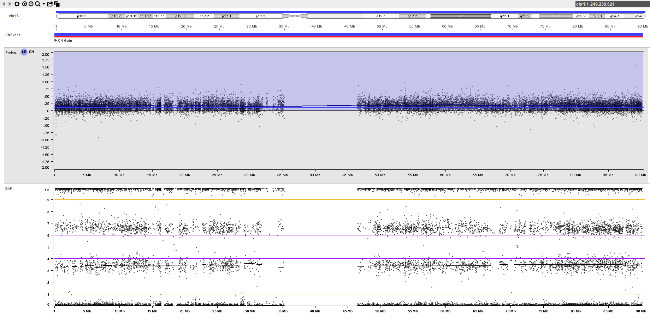		trisomy 16 in the early zygote / embryo 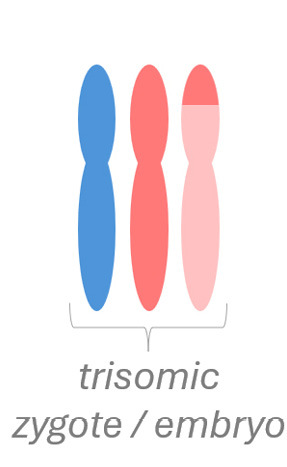	
long-term culture 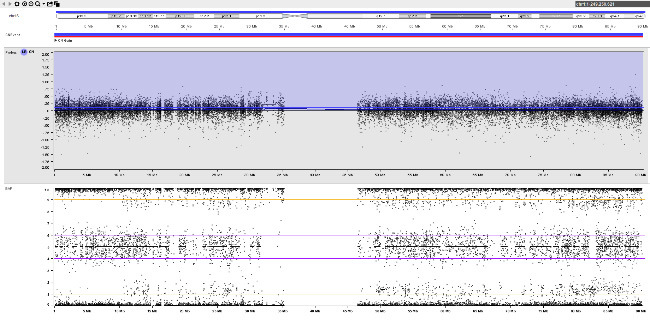		trisomy rescue in villus core 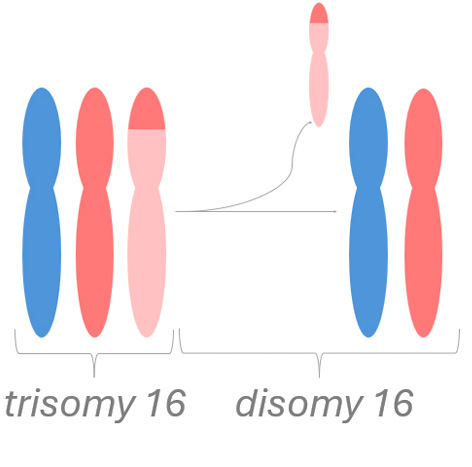	

**Table:** 

**B **			
fetal brain 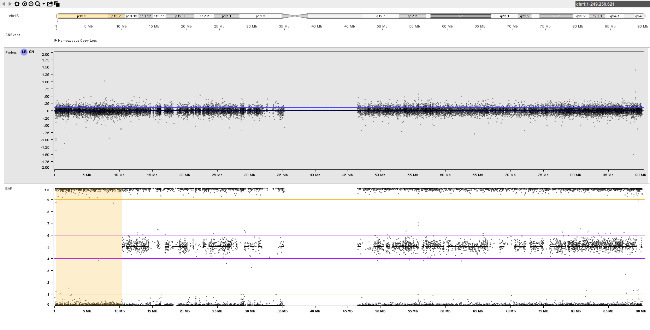		trisomy rescue in fetal tissue 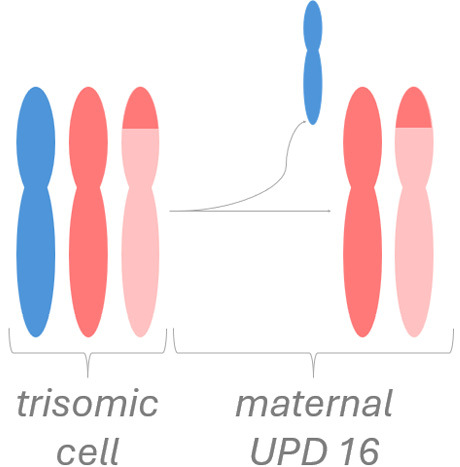	
fetal heart 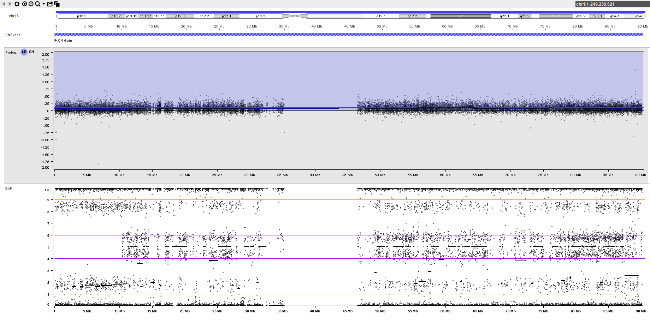			

**Figure 3:** Results of SNP-array analysis for chromosome 16, log R-ratio (top) and B-allele frequency (BAF) (bottom) are shown. (A) Apparently non-mosaic trisomy 16 in native villi and trisomy 16 mosaicism (~20 %) in long-term culture. Genotype (based on BAF in long-term culture and comparison to parental genotypes) indicates an origin of trisomy 16 in maternal meiosis I with normal biparental inheritance of chromosome 16 in disomic cells. (B) Normal copy number for chromosome 16 in brain. Trisomy 16 mosaicism in heart (~35 %). The genotype in fetal tissues indicates maternal UPD 16 with hetero- and isodisomic chromosomal segments. The isodisomic segment at 16p is secondary to recombination during maternal meiosis. (C) Illustration of the two separate trisomy rescue events in villus core and fetal tissue. Villus core: loss of one of the maternal chromosomes 16 (depicted in red and light red) leads to a normal karyotype with biparental inheritance. Fetal tissues: loss of the paternal chromosome 16 (blue) leads to maternal UPD 16.

This case illustrates the heterogeneous distribution of trisomic cells in different fetal and placental tissues which prevents a reliable prenatal prognosis and reflects the complex processes during early embryogenesis with two independent trisomy rescue events. 

## The impact of placental mosaicism on molecular karyotyping and the interpretation of NIPT results

In many laboratories, chromosomal microarray analysis (CMA) has replaced traditional karyotyping in prenatal diagnosis [51, 57]. Major advantages of CMA are a rapid turnaround time and increased resolution with a higher diagnostic yield [58–61]. By CMA, mosaicism may be detected down to a level of 10–20 % [35, 36]. To save time, DNA is preferably extracted from native chorionic villi without prior separation of trophoblast and villus core. However, native villi consist of both, trophoblast and mesenchymal cells, with a predominance of trophoblast, particularly in the villus tips [62]. This mixture of DNA from different cell populations may lead to misinterpretations in cases with mosaicism and a proper assignment to different types of CPM (or TFM if confirmed at amniocentesis) may not be possible without additional karyotyping or CMA on DNA extracted from cultured villi [63, 64]. This applies not only to large chromosome aberrations but also to rare cases of mosaicism for small copy number variations detected by CMA on DNA from native villi [51, 65–67].

Non-invasive prenatal testing (NIPT) is now widely used to screen for common aneuploidies and has renewed the interest in confined placental mosaicism [68]. The interpretation of NIPT results is not possible without understanding placental mosaicism. NIPT is based on cell free “fetal” DNA in maternal blood which in fact originates from apoptotic trophoblast cells [68–72]. Therefore, the terms “cell-free placental DNA” [68] or “cell-free trophoblast DNA” are more accurate. Results from NIPT essentially reflect the karyotype in trophoblast but not in villus core or fetal tissues and hence may lead to false positive findings in CPM I and III and false negative results in TFM V [17, 18]. Due to these constraints, abnormal NIPT results are not diagnostic but require confirmation by invasive testing and must be interpreted in conjunction with prenatal ultrasound findings.

## Outlook

Two recent studies applied genome sequencing to term and early human placentas, respectively [73, 74]. Both found a high load of postzygotic clonal mosaic variants/mutations across multiple sites of the placenta. In the study of Coorens et al. [73] the highest mutational burden was observed in trophoblast. These findings indicate that genetic mosaic variation is an inherent feature of placental tissue and extends far beyond classic chromosome aberrations [75].

While many of the sequence variants detected in the above-mentioned studies have a low variant allele frequency, caution is warranted when using placental DNA or cell free trophoblast DNA for prenatal testing of monogenic disorders [74].
